# A Case Report of Rapidly Lethal Acute Respiratory Distress Syndrome Secondary to Coronavirus Disease 2019 Viral Pneumonia

**DOI:** 10.7759/cureus.8228

**Published:** 2020-05-21

**Authors:** Mitchell K Ng, Jason Ngo, Anooj Patel, Drew Patel, Kenneth K Ng

**Affiliations:** 1 Orthopaedic Surgery, Maimonides Medical Center, Brooklyn, USA; 2 Anesthesiology, Saint Barnabas Medical Center, Livingston, USA; 3 Plastic Surgery, Case Western Reserve University School of Medicine, Cleveland, USA; 4 Internal Medicine, Case Western Reserve University School of Medicine, Cleveland, USA; 5 Anesthesiology, State University of New York Downstate Medical Center, Brooklyn, USA

**Keywords:** coronavirus disease (covid-19), acute respiratory distress syndrome, berlin criteria

## Abstract

As of April 2020, the coronavirus 2019 (COVID-19) pandemic has resulted in more than 210,000 deaths globally. The most common cause of death from COVID-19 is acute respiratory failure. We report the case of a 78-year-old female with a history of hypertension, cerebrovascular accident (CVA), type 2 diabetes mellitus, and sarcoidosis, who presented to the emergency department with one day of dyspnea. The patient experienced a rapid decline in respiratory function and was intubated in the intensive care unit (ICU), meeting the Berlin criteria for severe acute respiratory distress syndrome (ARDS). Chest radiography revealed diffuse bilateral coalescent opacities, and severe acute respiratory syndrome coronavirus 2 (SARS-CoV-2) RNA swab test was positive for COVID-19. The patient experienced acute kidney injury with uptrending creatinine levels and remained lethargic and unresponsive throughout her ICU stay, suggestive of potential hypoxic brain injury. In light of the patient’s poor clinical status, age, and significant comorbidities, prognosis was conveyed about medical futility and patient’s family agreed to terminal extubation and the patient expired peacefully, exactly one week from hospital admission. This case report highlights the speed at which severe ARDS can present and contribute to end-organ dysfunction in COVID-19 patients.

## Introduction

As of the end of April 2020, the coronavirus disease 2019 (COVID-19) pandemic has infected more than 3,058,000 people and resulted in more than 210,000 deaths worldwide [1]. It is well documented that patients who are older and/or with significant comorbidities are at particular risk [2]. Patients older than 75 years of age account for nearly half of all deaths (47%), and the mortality rate of infected patients older than 80 is 14.8% [3]. The most common cause of death is acute hypoxemic respiratory failure from acute respiratory distress syndrome (ARDS) [4].

ARDS is characterized by a diffuse pulmonary inflammatory reaction, most commonly in response to infection (sepsis, pneumonia) that leads to increased vascular permeability and capillary endothelial damage, leading to diffuse alveolar damage and leakage of protein-rich exudate [5]. In one retrospective cohort study of 191 COVID-19 patients by Zhou et al., after sepsis ARDS was the most frequently observed complication (31%, or 51/191 patients) developing at a median of 12 days after illness onset [2]. We describe a case of severe ARDS secondary to COVID-19 viral pneumonia which progressed to multisystem end-organ dysfunction. Despite prompt intubation upon admission and appropriate medical management, the severe ARDS had progressed to multiple organ failure and in light of medical futility, the patient was terminally extubated exactly one week from admission.

## Case presentation

**Figure 1:**
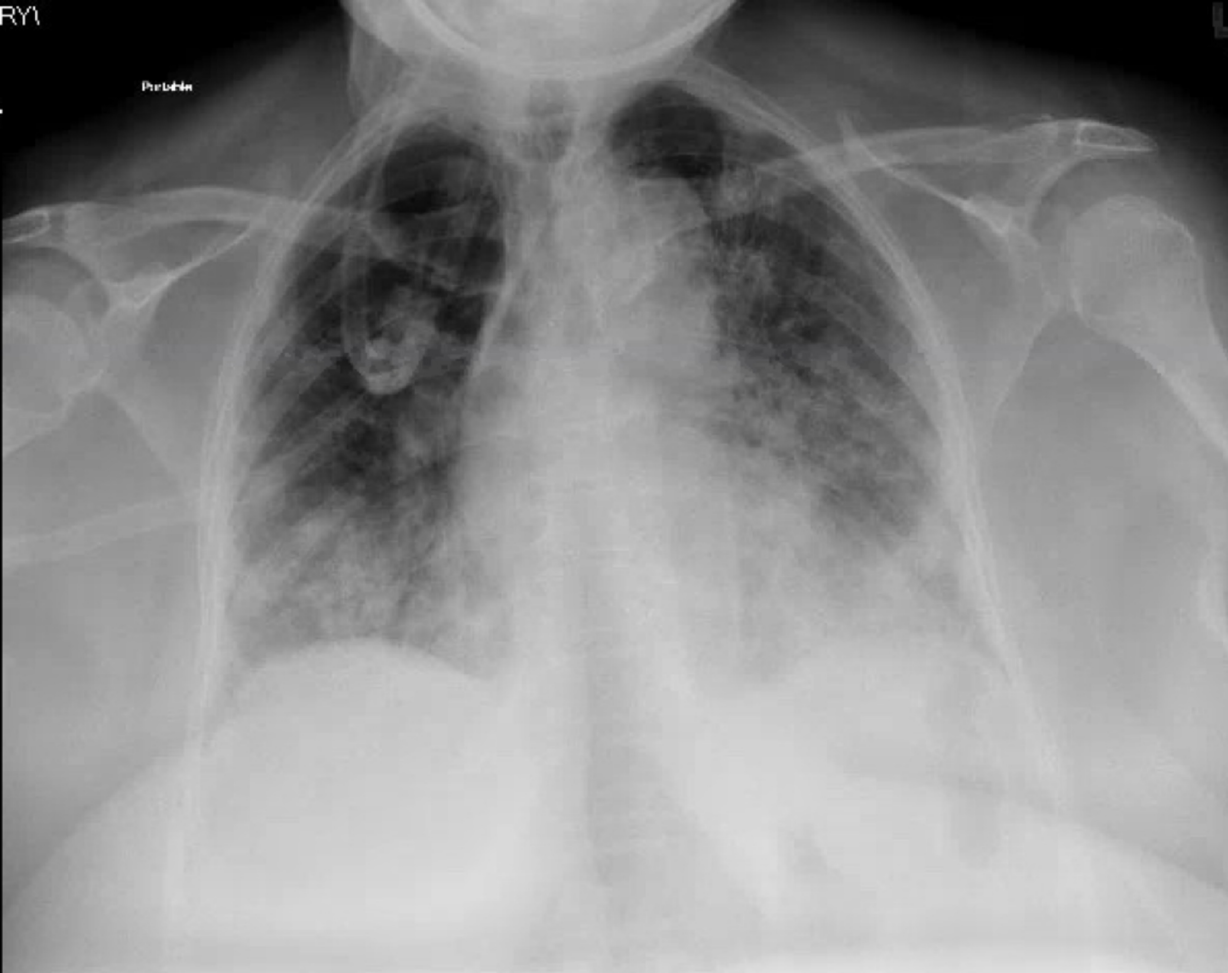
Initial emergency department chest radiograph reveals bilateral patchy opacities without cardiomegaly consistent with acute respiratory distress syndrome

A 78-year-old female with past medical history significant for hypertension, cerebrovascular accident (CVA), type 2 diabetes mellitus, and sarcoidosis presented to the emergency department with one day of shortness of breath. The patient reported dyspnea at both rest and upon exertion, she denied fevers/chills, chest pain, palpitations, upper respiratory symptoms (nasal congestion, rhinorrhea, nasal discharge), gastrointestinal symptoms (abdominal pain, constipation, diarrhea, changes in bowel habits), and 14-point review of systems otherwise negative. The patient was initially hypoxic with arterial oxygen level of 81%, which improved to 93% on 15 liters/minute (L/min) oxygen by a non-rebreather mask. Initial chest x-ray revealed diffuse bilateral patchy opacities without cardiomegaly consistent with ARDS, likely secondary to viral pneumonia (Figure 1).

**Figure 2:**
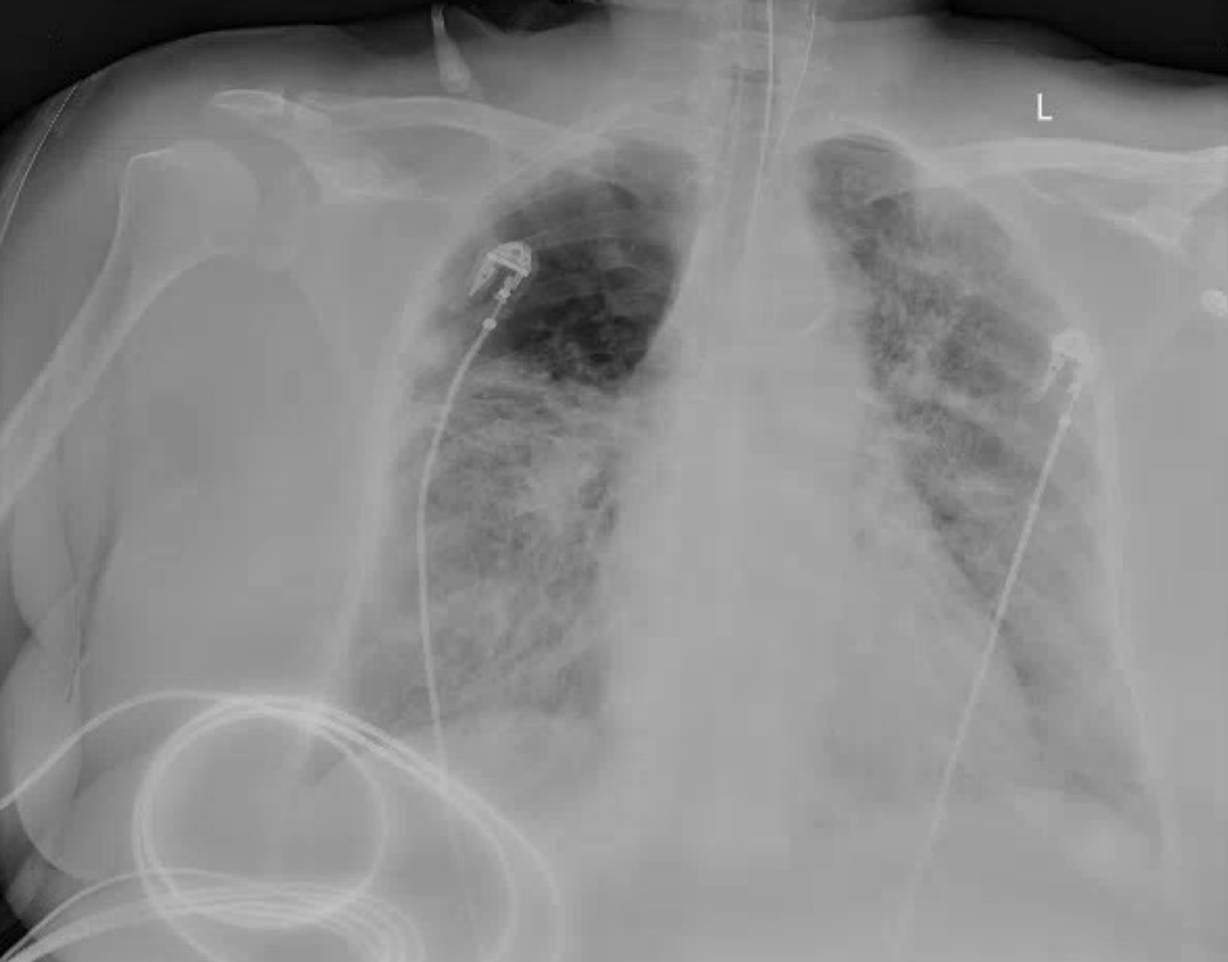
Repeat chest radiography reveals worsening diffuse bilateral patchy opacities and alveolar infiltrates

Due to impending respiratory failure, the patient was intubated and transferred to the intensive care unit (ICU) for further management. Of note, at this time the patient's arterial blood gas values were acidity level (pH) of 7.28, partial pressure of oxygen (PaO_2_) of 45 millimeters of mercury (mmHg), partial pressure of carbon dioxide (PaCO_2_) of 48 mmHg, bicarbonate level of 20 milliequivalents/liter, and oxygen saturation level of 88%. The patient met the Berlin criteria for severe ARDS, including ratio of PaO_2_ to fraction of inspired oxygen (FiO_2_) of 45 mmHg/60% of 75, which meets the <100 threshold characteristic of severe ARDS [2]. Management of the patient’s acute hypoxic respiratory failure secondary to viral pneumonia included severe acute respiratory syndrome coronavirus 2 (SARS-CoV-2) RNA throat swab test, which was positive for COVID-19, combination hydroxychloroquine and azithromycin per institution protocol, and continuation of continuous positive air pressure at 15 L/min. Repeat chest x-ray revealed worsening diffuse bilateral patchy opacities and alveolar infiltrates (Figure 2).

During the patient’s ICU stay, the patient was lethargic and unresponsive to verbal stimuli despite lack of sedation. She also experienced increased oxygen requirements and by day 2 of intubation was on maximal ventilator settings at tidal volume 400 mL, respiratory rate 25/minute, positive end-expiratory pressure (PEEP) 18 centimeters of water (cm H_2_O), and FIO_2_ 70%. The patient’s renal function continued to deteriorate (creatinine on admission 2.7 milligrams/deciliter (mg/dL) worsened to 5.1 mg/dL by ICU transfer) despite adequate fluid resuscitation and diuretics. 

The next day, in light of the patient’s poor clinical status, the prognosis was conveyed to family and the patient was made do-not-resuscitate with comfort care, and downgraded to general medicine floor. Due to the patient’s age and significant comorbidities, palliative care team spoke with family about medical futility (patient was unlikely to ever wean off ventilatory support, was unresponsive despite lack of sedation suggestive of hypoxic brain injury) and they agreed to withdrawal of care. One week from hospital admission, the patient was given multiple doses of morphine (total of 10 mg IV push) and midazolam (total of 5 mg IV push), taken off ventilatory support, and expired peacefully.

## Discussion

This case report highlights a case of rapidly progressive severe ARDS secondary to COVID-19 viral pneumonia, and serves as warning to clinicians about the speed at which respiratory failure with associated multiorgan dysfunction can occur. ARDS is one of the most frequently observed complications of COVID-19 [2]. Severe ARDS, classified by the Berlin criteria as PaO_2_/FiO_2_ < 100, is traditionally associated with 30%-40% mortality rate, but is associated with 52%-67% mortality in COVID-19 patients [5-7]. This stands in stark contrast with the overall mortality of COVID-19, which is estimated at 1%-2% [8]. One cohort study of 201 patients with COVID-19 pneumonia found risk factors associated with ARDS and progression from ARDS to death included older age, neutrophilia, and coagulation and organ dysfunction [9]. 

In this case, our patient experienced severe ARDS and subsequent multiorgan dysfunction. Apart from the acute hypoxic respiratory failure, our patient experienced acute kidney injury, a common complication seen in 29% of COVID-19 patients with ARDS. She also manifested neurological complications, which likewise commonly manifest in critically ill COVID-19 patients; in one case series, two-thirds of COVID-19 patients with ARDS experienced delirium/encephalopathy [10]. It is believed that systemic inflammatory dysregulation and/or impaired cerebrovascular hemodynamics contribute to cognitive decline during and after ARDS [10]. What is unique and informative about this case is the speed at which the dyspnea progressed to severe ARDS and ultimately multiorgan dysfunction, resulting in terminal extubation one week from admission. We believe this is most likely a result of the patient’s age and significant comorbidities, including HTN, CVA, type 2 diabetes mellitus, and sarcoidosis.

## Conclusions

This case report highlights the speed at which severe ARDS secondary to COVID-19 viral pneumonia can progress. The patient was terminally extubated one week from initial hospital admission in light of multiorgan system dysfunction, including rapidly declining respiratory function, acute kidney injury, and probable hypoxic brain injury. Further research is required to more fully characterize risk factors that contribute to poor prognosis.
